# Expression of S100A4, E-cadherin, α- and β-catenin in breast cancer biopsies

**DOI:** 10.1038/sj.bjc.6600624

**Published:** 2002-11-12

**Authors:** K B Pedersen, J M Nesland, Ø Fodstad, G M Mælandsmo

**Affiliations:** Department of Tumour Biology, The Norwegian Radium Hospital, Montebello, N-0310 Oslo, Norway; Department of Pathology, The Norwegian Radium Hospital, Montebello, N-0310 Oslo, Norway

**Keywords:** S100A4, mts1, calcium-binding protein, E-cadherin, immunohistochemistry, prognostic factor

## Abstract

In 66 breast cancer biopsies, the expression of the Ca^2+^-binding protein S100A4, E-cadherin, α- and β-catenin was examined by immunohistochemistry, and the results were related to clinical and pathological parameters. High levels of S100A4 were found to significantly correlate with histological grade (*P*=0.030) and loss of oestrogen receptor (*P*=0.046), but not to the time interval between surgery and development of distant metastasis (*P*=0.51) or to patient survival (*P*=0.89). Loss of E-cadherin expression, associated with altered cell–cell adhesion, showed a highly significant association to overall survival (*P*=0.020) and metastasis-free period (*P*=0.0052). In multivariate analysis, only lymph node involvement was a more significant predictor of patient demise. No association was found between expression of S100A4 and any single member of the cadherin–catenin complex, but a trend (*P*=0.053) towards reduced expression of one or several of these proteins and S100A4 immunoreactivity was observed. In conclusion, although our results suggest an association between S100A4 expression and an aggressive tumour phenotype, no relationship to overall survival was found. Deregulation of E-cadherin expression, however, was of high prognostic significance.

*British Journal of Cancer* (2002) **87**, 1281–1286. doi:10.1038/sj.bjc.6600624 www.bjcancer.com

© 2002 Cancer Research UK

## 

Breast cancer is the leading cause of cancer deaths among women in the Western world, with almost one woman in 11 developing cancer, and nearly half of these dying from metastatic disease. Identifying patients likely to develop metastatic spread at an early stage in disease progression may have important clinical implications and may affect treatment regimens, and several prognostic factors are established. Of these are lymph node involvement, tumour diameter and histological grade considered the most informative and hence commonly used in the clinical setting. Candidate molecular markers of breast cancer progression include c-*erb*B-2, ER, PgR, p53, integrins and proteases. However, to date none of these markers have turned out to add considerably to the prognostic significance of the clinical and pathological factors mentioned above (Walker *et al*, 1997).

The S100 family of Ca^2+^-binding proteins comprises 19 members, each member exhibiting a unique expression pattern in human tissues and exerting different functions (Donato, 2001). The S100A4 gene, cloned from murine mammary carcinoma cells, has been shown to be specifically expressed in cells with high metastatic capability (Ebralidze *et al*, 1989), but how S100A4 exerts its putative metastasis-promoting effects is largely unknown (Barraclough, 1998). The S100A4 protein has been found to colocalize with filamentous actin (Mandinova *et al*, 1998) and interact with nonmuscle myosin (Kriajevska *et al*, 1994) and nonmuscle tropomyosin (Takenaga *et al*, 1994), and therefore supposed to be involved in regulating cytoskeletal dynamics. We have previously shown, using a specific ribozyme transfected into highly metastatic S100A4 expressing human osteosarcoma cells, that reduced expression of S100A4 leads to a nearly complete reversal of the metastatic phenotype in an animal model (Mælandsmo *et al*, 1996). Correspondingly, the ribozyme-transfected cells were less motile and invasive *in vitro*, possibly due to deregulated expression of matrix metalloproteinases (MMPs) and tissue inhibitors of metalloproteinases (TIMPs) (Bjornland *et al*, 1999).

E-cadherin is a member of the cadherin superfamily that mediates homophilic, calcium-dependent cell–cell adhesion (Takeichi, 1991). Catenins, comprising at least three molecules, α-, β- and γ-catenin, are cadherin-associated intracellular proteins linking the cadherins to the actin cytoskeleton (Ozawa *et al*, 1989). In several types of carcinomas reduced expression or abnormal location of E-cadherin and the catenins have been observed, and the involvement of the E-cadherin–catenin system in suppressing cancer progression is well established (Bracke *et al*, 1996; Hirohashi, 1998; Behrens, 1999).

Recently, prognostic significance of S100A4 expression has been shown in several cancer types (Kimura *et al*, 2000; Rudland *et al*, 2000; Yonemura *et al*, 2000; Ninomiya *et al*, 2001). Surprisingly, for stage I and II breast carcinoma patients S100A4 was found to be the most significant predictor of patient survival, also compared to the well documented clinical and pathological parameters (Rudland *et al*, 2000). In other studies, an inverse correlation between S100A4 and members of the E-cadherin–catenin complex was revealed in non small cell lung cancer (NSCLC) and gastric cancer (Kimura *et al*, 2000; Yonemura *et al*, 2000). The possible association between these metastasis-associated proteins has not, to our knowledge, been studied in tumour biopsies from breast cancer patients. Moreover, the importance of E-cadherin in breast cancer is somewhat uncertain, and in different tumour panels it has been found that loss of, as well as sustained, E-cadherin expression was of prognostic significance (Lipponen *et al*, 1994; Siitonen *et al*, 1996; Bukholm *et al*, 1998; Tan *et al*, 1999; Gillett *et al*, 2001).

The main objective of the present study was to study the expression patterns of S100A4 and E-cadherin in a panel of 66 breast cancer biopsies and to investigate whether the expression levels were associated with known tumour variables or patient survival.

## MATERIALS AND METHODS

### Clinical specimens

Tumour tissue was collected with ethical approval and full informed consent at primary operation from 66 patients with operable breast cancer at the Norwegian Radium Hospital between 1992 and 1994. The fresh tumour tissue was snap frozen in liquid nitrogen and stored at −70°C. The surgical treatment given was either radical mastectomy (77%) or lumpectomy (23%) with or without sampling of axillary glands. Patients were assigned to chemotherapy, hormone therapy or radiation, or a combination of these, depending on the characteristics of the tumour. The lymph nodes, when available, were assessed histologically and recorded as containing or not containing carcinoma cells, and were not analysed with respect to the number of lymph nodes involved. Mean age at presentation was 55 years (range 32–80). Tumour diameter, histological grade, oestrogen and progesterone receptor status were recorded for all patients. Oestrogen and progesterone receptor status were measured using a ligand-binding assay (cut-off value 10 fmol/mg protein) or immunocytochemistry (>20% of the cells showing nuclear staining). An overview of the clinical and pathological characteristics is summarised in Table 1

**Table 1:**
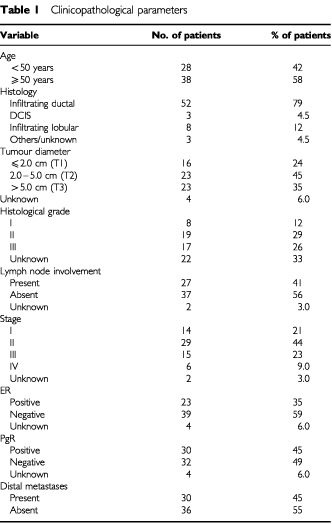
Clinicopathological parameters

.

### Antibodies

Polyclonal anti-S100A4 antibody was purchased from DAKO, Glostrup, Denmark and used in dilution of 1 : 50. Monoclonal anti-α-catenin (Santa Cruz Biotechnology, Santa Cruz, USA), anti-β-catenin (Transduction Laboratories, Lexington, USA) and anti-E-cadherin (Zymed, San Francisco, USA) were all diluted 1 : 200. The specificity of the S100A4 antibody is described elsewhere (Pedrocchi *et al*, 1994).

### Immunohistochemistry

Tumour tissue was embedded in Tissue-Tek O.C.T. Compound (Sakura Finetek, Zoeterwoude, The Netherlands), and 5 μm thin frozen sections were cut consecutively from the tumour samples and stored air-protected at −70°C. One section from each tissue sample was H&E-stained and examined for the presence of tumour tissue. Sections were fixed in acetone, blocked with normal rabbit serum (monoclonal antibodies) or normal horse serum (polyclonal antibodies) and incubated with primary antibody for 30 min at room temperature. For the monoclonal antibodies, antibody binding was detected using a secondary rabbit anti-mouse antibody followed by an alkaline phosphatase complex (DAKO, Glostrup, Denmark). Polyclonal antibody binding was detected using alkaline phosphatase-conjugated swine anti-rabbit (DAKO, Glostrup, Denmark) and rabbit anti-swine (ICN, Costa Mesa, USA) immunoglobulins. Bound antibody-alkaline phosphatase complex was visualised using New Fuchsin substrate. Sections were counterstained in Mayer's hematoxylin and mounted in Glycergel (DAKO, Glostrup, Denmark).

Sections were scored microscopically by two independent observers. For each sample, at least 100 cells, and usually more than 1000 cells, were analysed. For cases with regions of inhomogeneity within the same section, the total number of tumour cells was analysed. The number of immunopositive cells was semiquantitatively estimated: negative (−), 0%; borderline positive (+), 1–5%; moderately positive (++), 5–50%; and strongly positive (+++), >50% of the carcinoma cells stained. For S100A4 cytoplasmic staining was recorded, and for α-catenin, β-catenin and E-cadherin only membrane staining was regarded as positive. In addition, staining intensity was recorded for S100A4. The staining intensities reflected the number of immunopositive cells, thus conducting the analyses using this variable gave no significant associations not seen using the number of positive cells. Normal breast epithelium and lymphocytes adjacent to the tumour tissue served as positive control for the proteins of the cadherin–catenin complex and S100A4, respectively. Negative controls included substitution of the primary antibody with an unspecific antibody of the same subclass, as well as staining of cell lines previously known to be negative for S100A4, E-cadherin and the catenins.

### Statistical analysis

Follow-up information was obtained from the Norwegian Cancer Registry, a nation-wide register of all cancer patients, and from the patient records at the hospital. All data were updated to August 1, 2000, giving a mean follow-up for patients still alive of 79 months. The numbers in each of the four groups were too small for independent statistical analysis. Therefore, unless otherwise stated, immunohistochemically negative or borderline positive tumours were grouped as negative, and the moderately and strongly positive tumours were grouped as positive. The correlation of immunohistochemical staining of any of the proteins with each other or other tumour variables was tested using chi-squared tests. The other tumour variables tested included patient age, tumour diameter, lymph node status, histological grade and presence of ER or PgR in the primary tumour. For each of the proteins, association between staining and patient survival or time to development of distant metastases was tested using Kaplan–Meier survival plots. Patients dying from other causes than their breast cancer were excluded from the analysis. Multivariate analysis was performed using Cox proportional hazards regression model. Statistical analyses were performed using Statistical Package for the Social Sciences, version 6.1 (SPSS Inc., Chicago, USA).

## RESULTS

### Immunohistochemical staining

**Figure 1:**
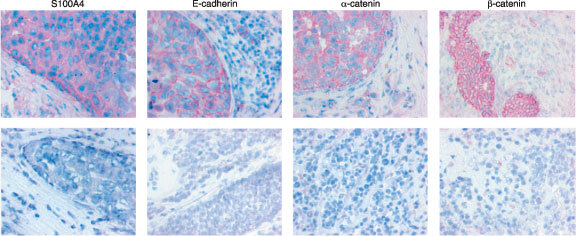
Top panel: Tumour tissue showing strong immunoreactivity (+++) to the proteins indicated. Note the cytoplasmic staining for S100A4, while the adhesion molecules show a membranous distribution. Lower panel: Tumour tissue showing no (−) immunoreactivity.

In normal breast tissue adjacent to the tumour S100A4 protein expression was detected in a variety of different cell types. The cytoplasm of smooth muscle cells of the vessel walls, lymphocytes, macrophages and the intralobular connective tissue were consistently positive, while the interlobular connective tissue showed no staining for S100A4. Epithelial cells of the ducts and ductules occasionally showed positive staining, though the majority of these were not immunoreactive. The staining of normal tissue was not assessed further, though it was observed that tumours expressing high amounts of S100A4 protein also showed stronger immunoreactivity in the corresponding normal tissue. Tumour cells displayed a uniform, cytoplasmic staining for S100A4 (Figure 1

). In 22 (35%) cases no immunoreactivity was observed, six (10%) samples were borderline positive, 16 (26%) samples were moderately positive and 18 (29%) were strongly positive.

Normal breast epithelium showed strong membrane immunoreactivity for E-cadherin, α- and β-catenin. In tumour cells, staining was confined not only to the membrane (Figure 1), but also present diffusely in the cytoplasm. No immunoreactivity was observed in the nuclei. Normally functioning cadherin and catenin molecules are associated with the cell membrane, therefore only membrane staining was interpreted as positive. A summary of the immunohistochemical analysis is presented in Table 2

**Table 2:**
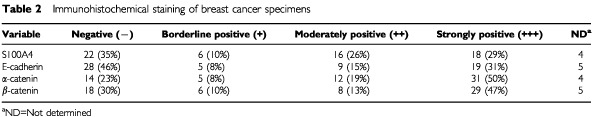
Immunohistochemical staining of breast cancer specimens

.

### Correlation of S100A4 protein expression with proteins in the cadherin–catenin complex

In the chi-square analysis, immunohistochemical staining for S100A4 was not correlated to expression of E-cadherin, α- or β-catenin. However, when the expression of these three interacting proteins was summarised as one variable, a trend towards reduced expression of one or several of these proteins and S100A4 immunoreactivity was seen (*P*=0.053). As expected, the proteins in the cadherin–catenin complex demonstrated a highly significant correlation to each other when analysed separately.

### Association between molecular markers andclinical parameters

**Figure 2:**
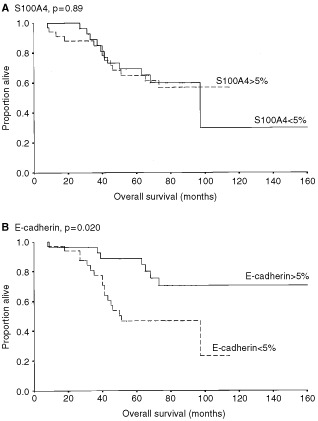
Kaplan–Meier survival curves demonstrating the relationship between patient survival and protein expression of S100A4 (a) and (b) E-cadherin.

In contrast to previous reports, in our study S100A4 immunoreactivity was not associated with overall survival or time to metastasis. (Kaplan–Meier survival plot, *P*=0.89 and *P*=0.51, respectively. See Figure 2

). Including only the ductal carcinomas (55 cases) in the analyses, or including the borderline group within the positive samples, did not in any case dramatically affect the analyses. S100A4 protein expression was significantly correlated to loss of ER (*P*=0.046) using chi-squared tests. Particularly the S100A4 negative group showed a clear tendency towards ER expression, with 20 of 27 (74%) samples positive for ER compared to 15 of 31 (48%) samples in the S100A4 positive group. S100A4 immunoreactivity demonstrated a significant correlation with high histological grade (*P*=0.030), with only five of 22 (23%) of the S100A4 negative tumours described as a grade III tumour, compared to 12 of 22 (55%) of the S100A4 positive tumours. S100A4 staining was not correlated to the patients' ages at time of presentation, PgR, lymph node involvement or tumour diameter.

Of the three other molecular markers examined, loss of E-cadherin membrane staining was associated with poor prognosis (*P*=0.020, Figure 2) and a short metastasis-free period (*P*=0.0052), while α- or β-catenin expression did not reveal any association with these parameters. Reduced expression of one or more of the examined proteins in the cadherin–catenin complex showed only a trend (*P*=0.055) towards shortened survival, and did not add prognostic information to the established associations for loss of E-cadherin staining. Of the other tumour variables tested, lymph node involvement (*P*=0.0047) and tumour diameter of more than 5.0 cm (*P*=0.0017) showed a significant association with shorter patient survival. In contrast, ER and PgR status, patient age and histological grade were of no prognostic significance in this subset of tumour samples. None of the E-cadherin-associated adhesion molecules tested showed any correlation to any of the tumour variables above.

Using the Cox regression model, nodal status proved to give the highest significance (*P*=0.0075), followed by loss of E-cadherin immunoreactivity (*P*=0.016) and tumour diameter of more than 5.0 cm (*P*=0.023). The multivariate analysis is summarised in Table 3

**Table 3:**
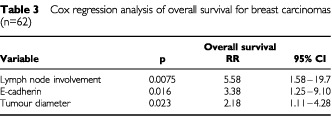
Cox regression analysis of overall survival for breast carcinomas (*n*=62)

.

## DISCUSSION

We examined by immunohistochemistry the protein expression of S100A4, a metastasis-associated protein, in human breast cancer specimens. In addition, expression of the cell–cell adhesion molecules E-cadherin, α- and β-catenin were investigated, and the protein levels of these markers were related to clinicopathological variables and survival data.

Of the 66 breast carcinomas in our tumour material, only 62 were available for analysis of S100A4 immunoreactivity. The expression differed widely, but positive cells showed a uniform cytoplasmic staining pattern. Twenty-eight cases (45%) were scored as negative and 34 cases (55%) as positive. In contrast to the recent report by Rudland *et al* (2000), we did not find any association between the expression levels of S100A4 and clinical outcome. In their study, S100A4 was the most significant predictor of patient survival in a panel of 349 stage I and II breast cancer patients. Several possible explanations for this inconsistency might exist, of which the most likely is that our panel includes fewer samples (*n*=62), and that we also included stage III and IV breast cancer patients in the study. One could speculate that the prognostic value of S100A4 might be more questionable in the more advanced cases. When excluding these patients, however, we got similar results, arguing against this being the reason for the different results in the two studies. It should be noted that the follow-up period in our study is relatively short, with a mean of 79 months for patients still alive. Moreover, we investigated snap-frozen, acetone-fixed tumour biopsies, while Rudland *et al* (2000) examined archival formalin-fixed, paraffin-embedded specimens. Such differences in preservation and fixation could possibly affect the results, although both we and others have previously obtained consistent results with anti-S100A4 antibodies regardless of storage conditions or fixation method (Rudland *et al*, 2000). Taken together, our results seem to question the prognostic value of S100A4 in breast cancer patients, and further investigations are warranted to elucidate the importance of this S100 protein as a prognostic factor.

In the present study, we included within an 18-month period all breast cancer patients undergoing surgery at our hospital, from whom it was possible to obtain a biopsy for research purposes. This explains the overrepresentation of patients with large tumours. As can be seen in the staging and clinical data presented in Table 1, biopsies from patients with stage I breast cancers are rather few compared to a normal stage distribution. Nevertheless, the fact that known prognostic parameters such as nodal status and tumour diameter were significantly associated with patient survival ensures the quality of our panel of tumour material.

Despite the lack of relation to survival, we found a significant association between high S100A4 protein expression and histological grade III (*P*=0.030), as well as an inverse correlation to expression of oestrogen receptor (*P*=0.046). These findings indicate a relation between S100A4 and an aggressive phenotype, as both loss of ER and high histological grade are known indicators of aggressive disease. The observed inverse correlation between S100A4 and ER is in agreement with previous expression studies performed on human breast cancer specimens (Albertazzi *et al*, 1998; Nikitenko *et al*, 2000). Additionally, transfection of mts1 (mouse S100A4) into human MCF-7 cells resulted in oestrogen-independent growth *in vivo* and deregulation of oestrogen-responsive genes (Grigorian *et al*, 1996).

A significant association was seen between loss of E-cadherin immunoreactivity at the cell membrane and both patient survival and metastasis-free period. In addition, loss of E-cadherin staining proved to be an independent prognostic factor using multivariate analyses, as did lymph node involvement and tumour diameter. This finding is in concordance with most previous studies (Lipponen *et al*, 1994; Siitonen *et al*, 1996), and strengthens the hypothesis of E-cadherin being a metastasis-suppressing molecule and a prognostic indicator in breast cancer. Two recent studies have highlighted the role of preserved E-cadherin membrane staining as an indicator of early patient demise (Gillett *et al*, 2001; Tan *et al*, 1999), and re-expression of E-cadherin also occurs in metastatic tumours (Bukholm *et al*, 2000). These reports indicate that retained adhesive ability may promote invasion, colony formation and growth in a secondary organ. Based upon our results, one might speculate that local invasion and extravasation is promoted through loss of E-cadherin function, and that the adhesive properties required for formation of metastases could possibly be regained through re-expression of E-cadherin or expression of other adhesion molecules. Additionally, in E-cadherin-negative tumours invasion could be promoted through ectodomain shedding (Noe *et al*, 2001). The HECD-1 antibody used here recognises the extracellular domain of human E-cadherin, thus E-cadherin negative tumours may have a proteolytically cleaved E-cadherin, lacking the shedded ectodomain. Hence, in these tumours invasion is possibly promoted through processes distinct from the mere loss of cell–cell adhesion.

An inverse correlation between S100A4 and members of the cadherin–catenin complex has recently been shown in gastric cancer and NSCLC (Kimura *et al*, 2000; Yonemura *et al*, 2000), and S100A4 and E-cadherin have been reported to be inversely regulated in a murine mammary carcinoma cell line (Keirsebilck *et al*, 1998). We did, however, not find any correlation between S100A4 immunoreactivity and expression of E-cadherin, α- or β-catenin, but a trend towards reduced expression of one or several of the proteins in the cadherin–catenin complex and S100A4 could be observed (*P*=0.053). The discrepancy with earlier results is possibly a reflection of different biological behaviour of tumours of different origin. Furthermore, the immunohistochemical evaluation may be critical, as we, in contrast to the study by Kimura *et al* (2000), have interpreted cytoplasmic staining of cadherins and catenins as negative because normally functioning adhesion molecules are located in association with the cell membrane.

Immunoreactivity to proteins in the cadherin–catenin complex was in our material observed at the cell membrane in both tumour and normal epithelial cells, as well as in the cytoplasm of some tumour cells, while no staining was observed in the nuclei. β-catenin is known to accumulate in the cytoplasm and nucleus of colorectal cancer cells through mutations in APC or β-catenin itself (Morin *et al*, 1997), and thereby function as a transcriptional activator when complexed with members of the Tcf family of DNA-binding proteins (Behrens *et al*, 1996). Nuclear accumulation of β-catenin has recently also been shown in breast cancer (Lin *et al*, 2000), while nuclear localisation of α-catenin has not been reported. In our panel of dry-frozen breast cancer biopsies, we did not detect any β-catenin in the nuclei. This is in agreement with Munne *et al* (1999), who, using the same antibody as in the present study, detected nuclear β-catenin in formalin-fixed, paraffin-embedded colorectal cancer sections, but not in adjacent frozen sections from the same tumour tissue. The reasons for this phenomenon are at present unknown, but it indicates that the COOH-terminal part of β-catenin is masked in frozen specimens, possibly due to interaction with other molecules. That β-catenin might be localised to the nucleus in some of the examined biopsies could therefore not be ruled out.

In conclusion, we have shown that loss of E-cadherin immunoreactivity was significantly associated with patient survival and tumour progression in the examined breast cancer specimens. Neither E-cadherin nor the catenins were associated with S100A4 expression, while a significant correlation was found between high levels of S100A4 and both histological grade III and loss of ER. Despite the association between aggressive tumours and S100A4 expression, the protein level was not a prognostic factor in this panel of breast cancer samples, and further studies are warranted to elucidate the prognostic role of S100A4.
